# Sartre’s Existential Psychopathology: The Role of Freedom and Personal History in Mental Health

**DOI:** 10.1192/j.eurpsy.2025.614

**Published:** 2025-08-26

**Authors:** F. Cunha, I. Santos, N. Castro, R. Andrade, S. Borges

**Affiliations:** 1 ULS Viseu Dão-Lafões, Viseu, Portugal

## Abstract

**Introduction:**

The existentialist approach to psychopathology emphasizes the need to understand the conditions that allow for the emergence of psychological disorders, referred to as “conditions of psychopathologization.” This perspective, rooted in phenomenological methodology, rejects deterministic biomedical models, favoring a holistic view that considers the subject’s lived experience, freedom, and choice. Drawing on the work of Jean-Paul Sartre, this study aims to analyze how existential conditions such as past events, personal choices, and social contexts contribute to the development of psychopathological states.

**Objectives:**

The primary objective of this research is to explore the existentialist understanding of psychopathology, particularly focusing on Sartre’s contributions to making psychopathological experiences comprehensible.

**Methods:**

Narrative review of relevant literature.

**Results:**

The findings highlight that psychopathological conditions often arise when individuals are alienated from their own projects of being. These conditions are shaped by personal histories, societal structures, and choices made in “bad faith” (self-deception). Past events, such as family dynamics, social oppression, and traumatic experiences, play a critical role in shaping the individual’s choices and actions. Sartre argues that when individuals distance themselves from their authentic desires and intentions they experience existential alienation, which manifests as psychopathology. A key result is the understanding that psychopathology should not be seen as a mere malfunction of the brain, as suggested by the biomedical model. Rather, it is a comprehensible event in the subject’s individual and social history. The deviation from an authentic life project is central to understanding the origin of psychological disorders.

**Conclusions:**

Sartre’s existentialist framework offers significant contributions to the field of psychopathology by making psychological suffering comprehensible through the lens of personal freedom and choice. The rejection of a purely biomedical or deterministic approach allows for a more nuanced understanding of the individual’s psychological struggles as part of their broader life context. Psychopathology is thus seen as a breakdown in the relationship between the subject’s life project and their historical or existential situation, where the individual either chooses to distance themselves from their authentic self or is forced to do so by external contingencies. Sartre advocates for a “reciprocal” clinical relationship that recognizes the patient’s subjectivity, transforming the clinical space into one of genuine listening and understanding. This existentialist clinical model focuses on the subject-in-situation.

**Disclosure of Interest:**

None Declared

